# NIF inhibits the ability of PMNs to produce inflammatory cytokines and reactive oxygen species in response to *C. albicans*

**DOI:** 10.1128/spectrum.01479-25

**Published:** 2025-08-21

**Authors:** Yi-yang Wang, Ping Li, Jing Huang, Ruo-yu Jiang, Cheng-long Zhu, Shan-quan Sun

**Affiliations:** 1 Chongqing Medical University-University of Leicester Joint Institute 12550, Chongqing, China; 2 Department of Anatomy, Chongqing Medical University 12550, , Chongqing, China; 3 Graduate School, Wannan Medical College, Wuhu, Anhui, China; 4 School of Anesthesiology, Naval Medical University, Shanghai, China; 5 Faculty of Anesthesiology, Changhai Hospital, Naval Medical University, Shanghai, China; 6 Department of Biochemistry and Molecular Biology, College of Basic Medical Sciences, Naval Medical University 12521, Shanghai, China; National Chung Hsing University, Taichung, Taiwan

**Keywords:** nifedipine, polymorphonuclear neutrophil, *C. albicans*, NF-κB phosphorylation, antifungal immunity

## Abstract

Invasive fungal infections are associated with high mortality rates. The proliferation of *C. albicans* is mainly limited by the innate immune system, in which phagocytic cells, especially polymorphonuclear leukocytes (PMNs), play an important role. Several studies have shown that antihypertensive drugs can cause an increased risk of infection, but whether antihypertensive drugs affect the killing activity of PMNs against *C. albicans* has not been studied. Here, we demonstrated that nifedipine (NIF) inhibits neutrophil killing of *C. albicans*. NIF reduces the production of reactive oxygen species (ROS) and pro-inflammatory cytokines in PMNs by inhibiting the phosphorylation of NF-κB, thereby inhibiting killing of PMNs against *C. albicans*. Next, using the *C. albicans* injection mouse model of fungal sepsis, we observed NIF can exacerbate infection in mice. Finally, our study identified patients with fungal infections from the Medical Information Mart for Intensive Care (MIMIC-IV) database, and analysis of the data showed that NIF led to increased length of stay. In summary, we have found that the antihypertensive drug NIF can negatively regulate antifungal immunity by inhibiting the phosphorylation of the PMNs transcription factor NF-κB.

**IMPORTANCE:**

In this study, we demonstrated that NIF inhibited the ability of neutrophils to kill *C. albicans* by suppressing NF-κB phosphorylation, thereby reducing the production of ROS and pro-inflammatory cytokines in neutrophils. Furthermore, analysis of the MIMIC database revealed that NIF prolonged hospitalization duration in patients with fungal infections. Collectively, our findings suggest that antihypertensive drugs may exert adverse effects on the treatment of fungal infections, which could provide new insights for optimizing therapeutic strategies in clinical practice.

## INTRODUCTION

Fungal infections are a growing threat to global public health, and of the more than one million fungal species that have been discovered, several hundred can cause disease in humans (1). Pathogenic fungal infections infect hundreds of millions of patients globally each year, causing at least 1.5 million deaths annually (2). Invasive fungal infections (IFIs) are defined as systemic infections in which yeast or mold forms in deep tissues (3). Unlike superficial fungal infections, IFIs are a deadly disease with high morbidity and mortality rates, with a mortality rate of up to 50%, even with the application of current clinical guidelines, due to the increasing toxicity and resistance of existing drugs (4). *C. albicans* is the most common fungal pathogen, accounting for more than half of invasive candidiasis (5). Studies have shown that invasive *C. albicans* infections typically occur in the elderly and in patients with autoimmune deficiencies (6). Hypertension, the most prevalent chronic non-communicable disease globally, represents the primary risk factor for cardiovascular mortality in both urban and rural populations worldwide (7). Antihypertensive therapies typically necessitate lifelong administration. Among them, nifedipine (NIF) may impair neutrophil function and immune responses, potentially exerting harmful effects in conditions, such as cardiac dysfunction (8), cancer (9), and coronavirus disease (10). However, whether antihypertensive medications interfere with the immune system’s ability to combat *C. albicans* remains unclear.

Neutrophils (PMNs) are key members of host innate immunity and play an important role in clearing invading *C. albicans* (11). They are the most rapidly recruited immune cells to sites of injury and inflammation and play an important role in the immune system (12, 13). Upon *C. albicans* invasion, epithelial cells and macrophages release chemokines. These attract PMNs from the bloodstream to the infected tissue. There, they inhibit and clear *C. albicans* using various effector functions (14). The mechanism of fungal killing by PMNs consists mainly of oxidative and non-oxidative stresses. In terms of oxidative stress, PMNs recruited to the infection site release reactive oxygen species (ROS) and nitric oxide (NO) through two oxidative systems: reactive oxygen intermediates and reactive nitrogen intermediates (RNI) (15, 16). NO alone lacks direct antifungal activity. Instead, it reacts with superoxide to form peroxynitrite, a potent oxidizing agent that effectively kills *C. albicans* (17). On the contrary, PMNs can form neutrophil extracellular traps (NETs) to capture the *C. albicans*, inhibit its proliferation and multiplication, and release fungicide molecules, such as inflammatory cytokines and antimicrobial peptides, to destroy the fungal cell wall and cell membrane, then lead to the death of *C. albicans* (18). Activated PMNs release a series of cytokines, such as IL-1β, IL-6, TNF-α, IL-17, and IFN-γ, which further recruit circulating PMNs to the site of infection to kill *C. albicans* (19, 20).

In this study, representative common antihypertensive drugs were screened. Results showed that the calcium channel blocker NIF inhibited PMN-mediated killing of *C. albicans*. Therefore, we investigated the mechanism by which NIF suppresses this PMN function. Our study shows that during *C. albicans* infection, NIF inhibits NF-κB phosphorylation in PMNs. This action reduces oxidative stress, cuts down pro-inflammatory cytokine release, and weakens PMN-mediated *C. albicans* killing.

## MATERIALS AND METHODS

### Mice

C57BL/6J mice aged 6–8 weeks were purchased from Shanghai Slaughter Laboratory Animal Co. They were fed at the Institute of Immunology, Naval Medical University. All experimental procedures were performed in accordance with the standards of the Association for Assessment and Accreditation of Laboratory Animal Care (AAALAC).

### Reagents and antibodies

All the detailed information on the reagents are listed in Table S3. All the detailed information on the antibodies are listed in Table S4.

### Culture and heat inactivation of *C. albicans* strain SC5314

*C. albicans* strain SC5314 was provided by Dr. Ning Ma (Naval Hospital 905, Shanghai, China). Single colonies of *C. albicans* strain SC5314 from yeast-peptone-dextrose (YPD: LA0220, Solarbio) agar plates were inoculated into YPD medium by culture at 30°C overnight. Yeast cells were cultured for 3 h at 37°C in YPD medium plus 10% FBS to produce hyphae. Yeast or hyphae cells were washed three times and resuspended in PBS buffer, then incubated at 65°C for 1 h to kill the cells, then *C. albicans* yeast (HKCA-Y) or heat-killed *C. albicans* hyphae (HKCA-H) was obtained. Cells were plated on YPD agar plates to verify the death of heat-killed yeast or hyphal cells.

### Systemic *C. albicans* infection

For each infection, one colony was isolated from an agar plate and grown in YPD media at 30°C for 24 h. The yeast cells were washed three times in PBS buffer and diluted to 4 × 10^5^ yeast cells in 0.1 mL PBS buffer. Yeast cells were injected intravenously through the tail vein into mice. Weight loss was recorded every day. After 5 days, the kidney, liver, lung, and spleen were collected, and fungal burden was measured. Homogenized tissues were appropriately diluted and plated on YPD agar. Fungal colony-forming units (CFU) were counted after 24 h (21).

### Histopathology

For histological analysis, the kidneys were fixed in 4% paraformaldehyde solution for 2–3 days, and then handed over to Servicebio (Wuhan, China), embedded in paraffin, and sectioned. Next, 3 μm-thick sections were stained with hematoxylin and eosin (H&E), periodic-acid-Schiff (PAS), or anti-Ly-6G.

### Cell culture

Peripheral blood samples were collected from healthy volunteers recruited at Changhai Hospital (with approval from the hospital’s ethics committee). Human PMNs were purified by density gradient centrifugation with 3% Dextran and Ficoll-Hypaque solution (Millipore Sigma, Cytiva). At a concentration of 10^6^ cells/mL, the PMN pellet was resuspended in DMEM supplemented with 10% FBS, 1% glutamine, and 1% penicillin/streptomycin solution (22). PBMCs were isolated using lymphocyte separation medium (Corning, Manassas, VA) by employing density gradient centrifugation, adhering strictly to the manufacturer’s guidelines. At a concentration of 2 × 10^6^ cells/mL, PBMCs were resuspended in RPMI-1640 supplemented with 10% FBS, 1% glutamine, and 1% penicillin/streptomycin solution. The RAW264.7 mouse macrophage cell line was sourced from QuiCell Biotechnology, cultured in DMEM supplemented with 10% FBS, 1% glutamine, and 1% penicillin/streptomycin solution.

### ELISA

Concentrations of IL-1β, IL-6, and TNF-α in cell culture supernatants, mouse serum, or tissue homogenates were determined by ELISA kits (Elabscience) according to the manufacturer’s instructions.

### Immunofluorescence staining

For immunofluorescence staining, cells were fixed with paraformaldehyde (4%, Beyotime) for 20 min, then permeabilized with Triton X-100 (0.1%, Beyotime) for 20 min and blocked in 1% BSA (Beyotime) for 1 h at room temperature. The sections were then incubated with primary antibodies (1:200, anti-Cit-H3, ab5103, Abcam, USA; 1:200, anti-MPO, ab208670, Abcam, USA) overnight at 4°C. After three washes with PBS, sections were incubated with secondary antibodies (1:1,000, Goat Anti-Rabbit IgG, ab6721, Abcam, USA) for 1 h at room temperature. As a nuclear counterstain, we used DAPI (Beyotime).

### Western blotting

The denatured protein samples were separated by 10% SDS-PAGE gels and transferred to polyvinylidene fluoride (PVDF) membranes (Merck). The transferred membranes were blocked with blocking solution (Thermo) and incubated with primary antibody overnight at 4°C. The HRP-conjugated secondary antibody was then added to the washed membrane and shaken for 2 h at room temperature. Finally, we used ECL reagent (Beyotime) to visualize the membranes.

### *In vitro* killing assay

For the *in vitro* fungal killing assay, PMNs (5 × 10^5^ cells/well) were incubated with live *C. albicans* (Multiplicity of Infection, MOI = 3) for 3 h. After coculture, cells were washed with 1× PBS three times and then resuspended in fresh medium containing amphotericin B (MCE) at a final concentration of 30 µg/mL and cultured for 3 h at 37°C. Cells were washed again three times in 1× PBS and were then lysed by the addition of 0.02% Triton X-100 (Beyotime), and 100 mL of the suspension (1:1000 dilution) was spread on YPD plates. After incubation at 37°C for 24 h, killing was evaluated by counting the Candida colonies (23).

Purified immune cells were plated in triplicate (5 × 10^5^ cells/well) in 12-well plates and incubated overnight at 37°C with *C. albicans* (MOI 1:50 or 1:100) before fixing with 2% paraformaldehyde. *C. albicans* was stained with crystal violet (Beyotime). Percentages of fungal growth were quantified using ImageJ software (NIH) (20).

### ROS production

Purified PMNs were labeled with dihydrorhodamine (Beyotime) for 5 min before stimulation with HKCAs (MOI = 3) for 1 h at 37°C. Fluorescence microscopy photographs were taken, and ROS generation was quantified using ImageJ.

### Nitric oxide concentration assay

The indicated cell culture supernatants were collected and the amount of NO produced in the supernatants was assayed using a Nitric Oxide Assay Kit (Beyotime).

### Statistical analysis

Data are given as the mean ± SD. Figures and statistical analyses were generated using

GraphPad 8.0. Comparison of means to identify differences between two groups was performed using Student’s unpaired *t*-test. One-way analysis of variance (ANOVA), followed by Tukey’s post-hoc test, was used to compare normally distributed continuous data between multiple groups (>2). The log-rank (Mantel-Cox) test was applied for the survival curves. Two-way ANOVA, followed by Tukey’s post-hoc test was applied for the weight loss curves. *P* < 0.05 was considered significant.

## RESULTS

### NIF inhibits the ability of immune cells to kill *C. albicans*

**Fig 1:**
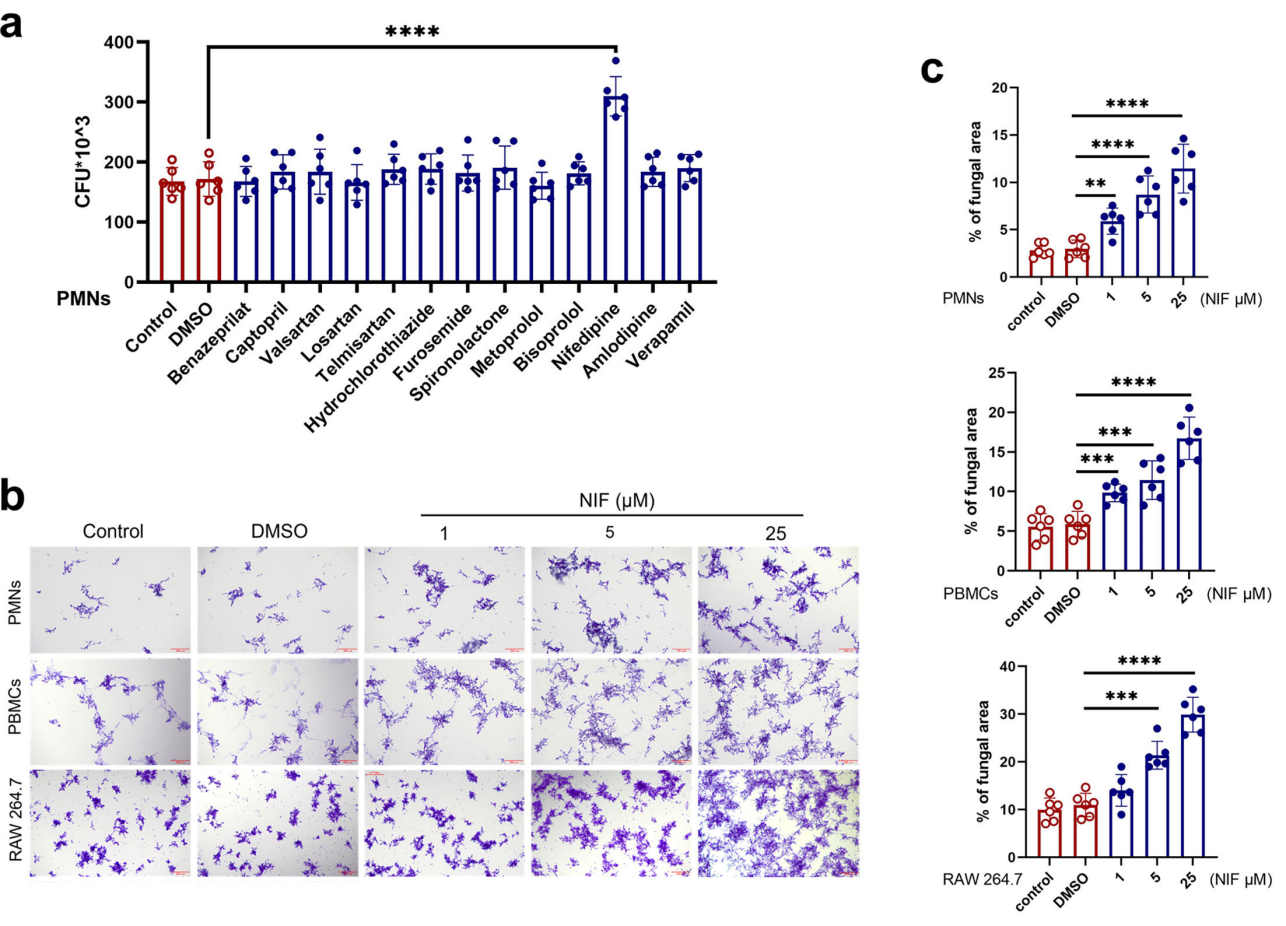
Nifedipine (NIF) inhibits the ability of immune cells to kill *C. albicans*. (**a**) After drug pretreatment of PMNs for 1 h, *C. albicans* (MOI = 3) was added and phagocytosed for 3 h. Amphotericin B (30 µM) was added and incubated for 3 h. Cells were lysed, and plates were coated with a certain concentration of dilution to count the number of colonies (*P* < 0.0001). (**b**) Killing capacity of PMNs, PBMCs, and RAW 264.7 as assessed by overnight coculture with *C. albicans* (MOI = 1:200). *C. albicans* were counterstained with crystal violet. (**c**) Six fields of view in each group and statistically analyzed using ImageJ. Scale bars: 200 µm (top: *P* = 0.0062, *P* < 0.0001, *P* < 0.0001; middle: *P* = 0.0006, *P* = 0.0004, *P* < 0.0001; bottom: *P* = 0.0002, *P* < 0.0001). Data are shown as mean ± SD. In a and c, **P < 0.01, ***P < 0.001, and ****P < 0.0001 (one-way analysis of variance in a and c). *n* = 6 per group. Assays were performed in triplicate.

We screened the following representative drugs from five classes of clinical first-line antihypertensive drugs (24): (i) angiotensin-converting enzyme inhibitor (ACEIs): benazepril, captopril; (ii) angiotensin receptor blocker (ARBs): valsartan, losartan, telmisartan; (iii) diuretics: hydrochlorothiazide, furosemide, spironolactone; (iv) beta blockers: metoprolol, bisoprolol; and (v) calcium channel blocker (CCB): NIF, amlodipine, and verapamil. PMNs are the most effective effector cells for killing *C. albicans*, which are also the only immune cells that can inhibit the transformation of yeast fungi into hyphae (11), so we chose PMNs as the main cells for study, and PBMCs and RAW264.7 were used for validation. We explored the effects of the above drugs on the killing capacity of PMNs by *in vitro* killing assay. The results showed that colony growth was significantly increased in the NIF-treated group. In contrast, other drugs including amlodipine and verapamil, despite also being CCBs, exhibited no significant effect on colony growth (Fig. 1a). Consequently, we focused exclusively on investigating NIF’s impairment of immune cell-mediated *C. albicans* killing capacity (Fig. 1a).

Furthermore, we need to exclude the direct effects of NIF on PMNs and *C. albicans*. After co-culture of different concentrations of NIF with PMNs and *C. albicans* for different times *in vitro*, CCK-8 assays demonstrated that 125 µM NIF exerted no significant effects on the viability of either PMNs or *C. albicans* at 3 or 6 h post-treatment. Prolonged incubation (12 h) revealed a safe concentration window for NIF at 25 µM (Fig. S1). We, therefore, hypothesize that NIF promotes *C. albicans* growth by impairing PMNs’ fungicidal activity against the pathogen. Consistent with this hypothesis, colony growth exhibited a dose-dependent inhibition proportional to increasing concentrations of NIF (1, 5, 25 µM), with statistically significant differences compared to the control group. This indicates NIF’s dose-dependent suppression of PMN-mediated killing of *C. albicans*. A similar inhibitory effect was also observed in PBMCs and RAW 264.7 cells (Fig. 1b and c). Collectively, these data indicate that NIF compromises the antifungal capacity of immune cells against *C. albicans*.

### NIF inhibits *C. albicans*-stimulated production of inflammatory cytokines and reactive oxygen species by PMNs

**Fig 2:**
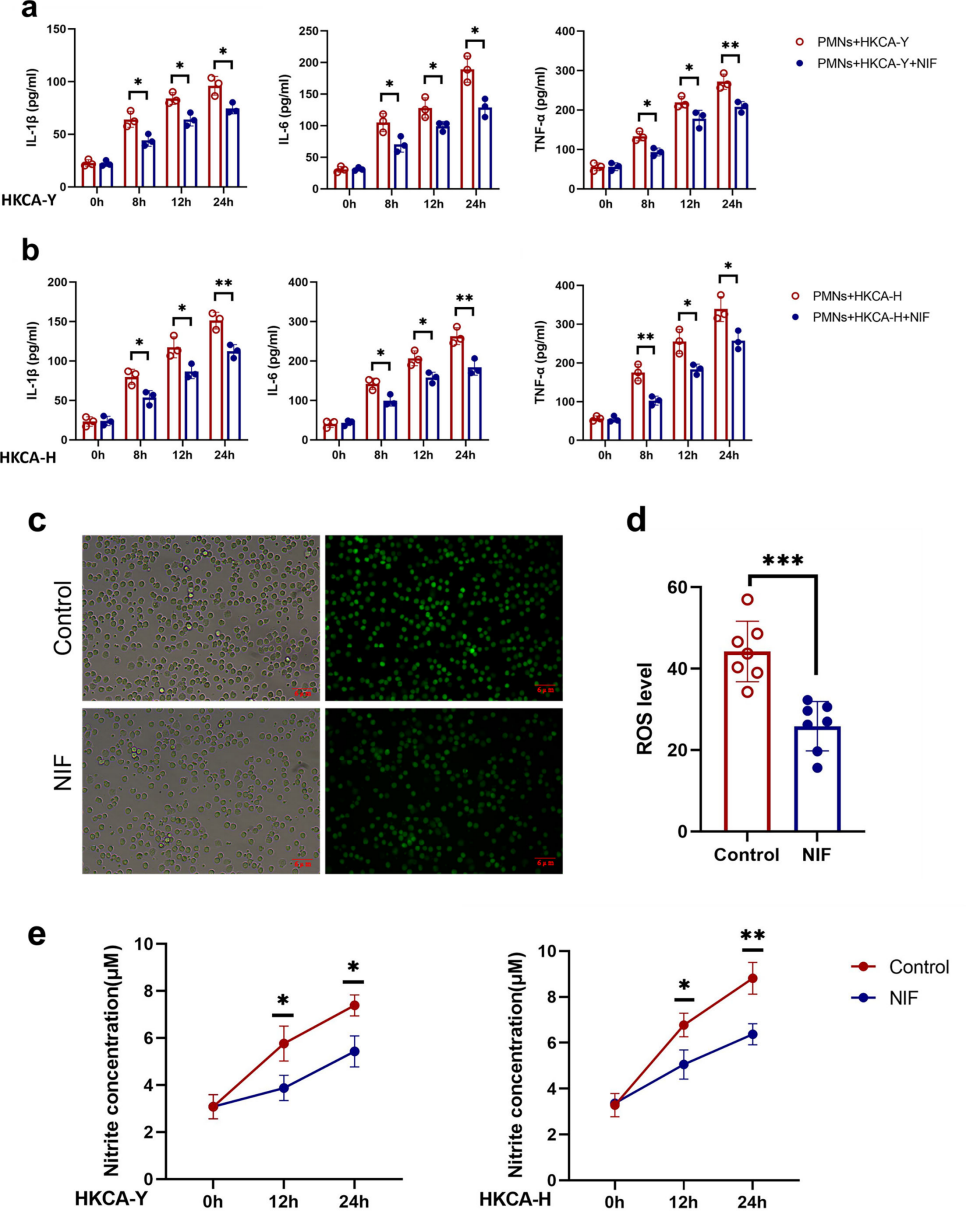
NIF inhibits *C. albicans*-stimulated production of inflammatory cytokines and reactive oxygen species in PMNs. (**a, b**) ELISA analysis of IL-1β, IL-6, and TNF-α in supernatants of PMNs stimulated with HKCA-Y (MOI = 10) (left: *P* = 0.023, *P* = 0.021, *P* = 0.032; middle: *P* = 0.031, *P* = 0.028, *P* = 0.017; right: *P* = 0.026, *P* = 0.036, *P* = 0.0072) (**a**) or HKCA-H (MOI = 10) for the indicated time points (left: *P* = 0.015, *P* = 0.032, *P* = 0.0051; middle: *P* = 0.021, *P* = 0.019, *P* = 0.0026; right: *P* = 0.0038, *P* = 0.016, *P* = 0.022) (**b**). (**c**) PMNs were stimulated with live *C. albicans* (MOI = 3) for 30 min, followed by measurement of ROS production. (**d**) Six fields of view in each group and statistically analyzed using ImageJ. Scale bars: 6 µm (*P* = 0.0003). (**e**) PMNs were stimulated with HKCA-Y (MOI = 10) and HKCA-H (MOI = 10) for the indicated times, followed by measurement of NO production (left: *P* = 0.033, *P* = 0.028; right: *P* = 0.026, *P* = 0.0043). Data are shown as mean ± SD. In a, b, d, and e, *P < 0.05, **P < 0.01, and ****P < 0.0001 (Student’s *t*-test in a–e). *n* = 3-6 per group. Assays were performed in triplicate.

Activated PMNs release a series of cytokines, such as IL-1β, IL-6, and TNF-α, which further recruit circulating PMNs to the infected area for killing fungal. To explore the mechanism of action of NIF, PMNs were stimulated using HKCA-Y and HKCA-H, and the production of inflammatory cytokines was detected by ELISA. We found that PMN production of pro-inflammatory cytokines (including IL-1β, IL-6, and TNF-α) was significantly reduced in the NIF-treated group compared to the control group at 8, 12, and 24 h under both HKCA-Y and HKCA-H infection conditions (Fig. 2a and b).

ROS and NO are the main effectors of neutrophil killing of *C. albicans* by oxidative stress. ROS and NO production in *C. albicans*-stimulated PMNs under NIF treatment was investigated using specific detection kits. Immunofluorescence assays demonstrated that NIF significantly inhibited ROS generation in PMNs following *C. albicans* stimulation compared to the control group (Fig. 2c and d). Furthermore, NO production by PMNs was reduced in the NIF-treated group compared to controls at 12 and 24 h under both HKCA-Y and HKCA-H infection conditions (Fig. 2e). PMNs *in vivo* can also rapidly release NET to prevent systemic fungi dissemination, which can be detected by assaying myeloperoxidase (MPO) and histone (CitH3) release. However, the results showed that the production of NETs was not significant in either the control or NIF group, suggesting that NIF could not enhance the resistance of *C. albicans* to neutrophil killing activity by inhibiting the production of NETs (Fig. S2). These findings collectively indicate that NIF suppresses PMN-mediated killing of *C. albicans* primarily by inhibiting the production of inflammatory cytokines, ROS, and NO in PMNs.

### NIF inhibits phosphorylation of the transcription factor NF-κB in PMNs

**Fig 3:**
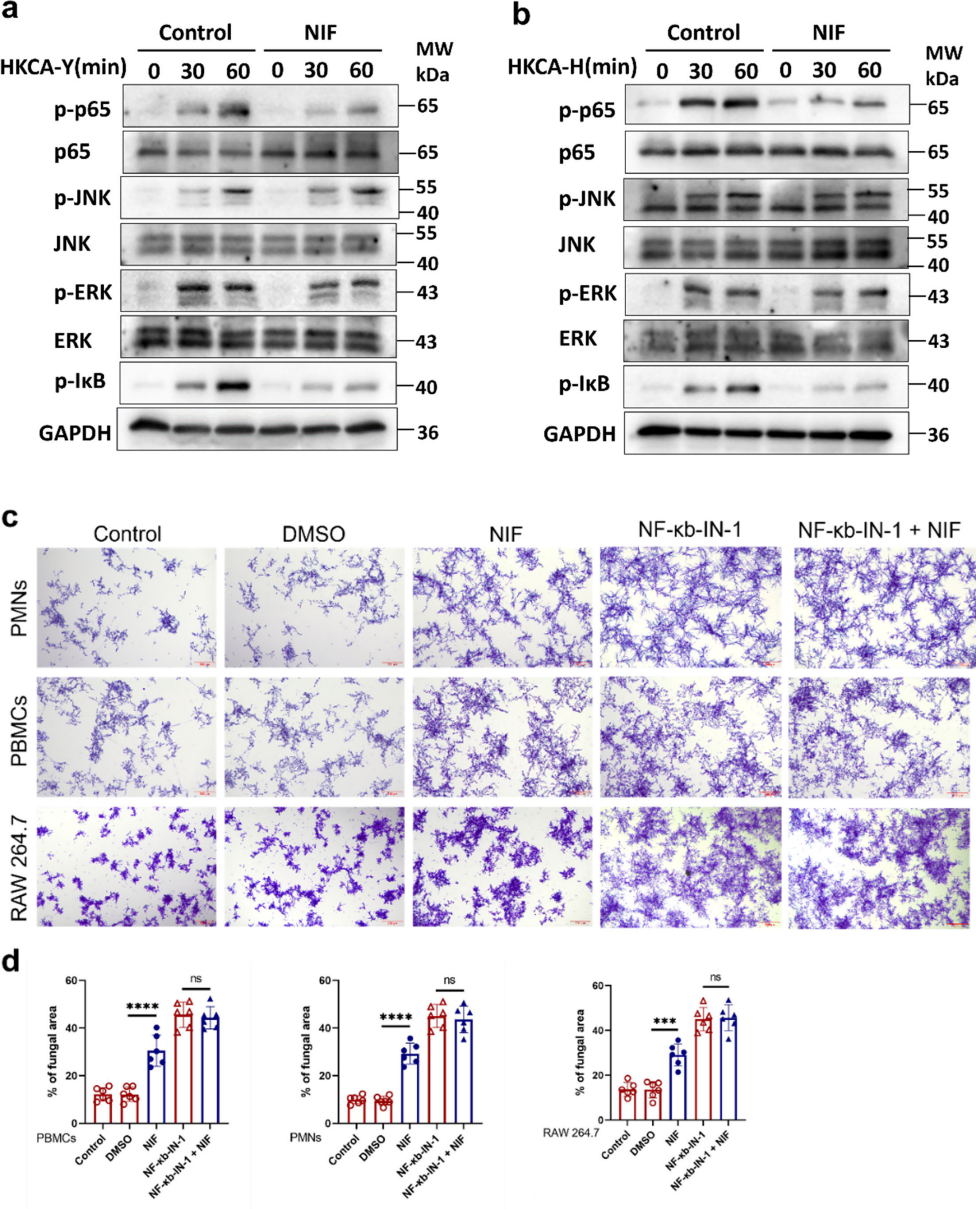
NIF inhibits phosphorylation of the transcription factor NF-κB in PMNs. (**a, b**) Western blot analysis of phosphorylated and total proteins in lysates of PMNs stimulated with HKCA-Y (MOI = 10) (a) or HKCA-H (MOI = 10) (b) for indicated time points. (**c**) Killing capacity of PMNs, PBMCs, and RAW 264.7 as assessed by overnight coculture with *C. albicans* (MOI = 1:200). *C. albicans* were counterstained with crystal violet. (**d**) Six fields of view in each group and statistically analyzed using ImageJ. Scale bars: 200 µm. (*n* = 6 per group) (left: *P* < 0.0001; middle: *P* < 0.0001; right: *P* = 0.0005). Data are shown as mean ± SD in d. ***P < 0.001, ****P < 0.0001, ns = not significant (one-way analysis of variance in d). Assays were performed in triplicate.

NF-κB, JNK, and ERK are important intracellular transcription factors. After *C. albicans* stimulates PMNs, it transmits signals into the cell through pattern recognition receptors on the cell surface, and these transcription factors are phosphorylated into the nucleus to promote the expression of relevant inflammatory factors (25). After pretreatment with DMSO and a final concentration of 10 µM NIF for 1 h in the control and NIF groups, respectively, the cells were stimulated with HKCA-Y and HKCA-H. Cells were collected at the indicated time points (0, 30, and 60 min), and changes in the total protein and phosphorylation levels of p-62, JNK, and ERK, as well as changes in the phosphorylation level of IκB, were detected. The results (Fig. 3a and b) showed that transcription factors were activated by phosphorylation under the stimulation of HKCA-Y and HKCA-H. The phosphorylation levels of p-62 and IκB were significantly reduced under the effect of NIF with no significant change in the total protein, and there were no significant changes in the total protein and phosphorylation levels of JNK and ERK.

To further verify the conclusion that NIF confers more resistance of *C. albicans* to neutrophil killing activity by inhibiting the phosphorylation of NF-κB in PMNs, we used NF-κB phosphorylation inhibitor NF-κB-IN-1 to co-treat PMNs with NIF, and (26) live *C. albicans* (MOI = 1:50) were added to PMNs in all groups after 1 h of drug pretreatment. *In vitro* killing experiments have shown (Fig. 3c and d) that the difference in immune cell killing ability caused by NIF was eliminated in the presence of NF-κB-IN-1.

### NIF inhibits killing of *C. albicans* by immune system *in vivo*

We have demonstrated that NIF inhibits the phosphorylation of NF-κB in PMNs during the antifungal process *in vitro*. Whether NIF also inhibits the process of immune response against *C. albicans in vivo* needs to be verified by constructing an animal model of invasive fungal infection using *C. albicans*-infected mice.

**Fig 4:**
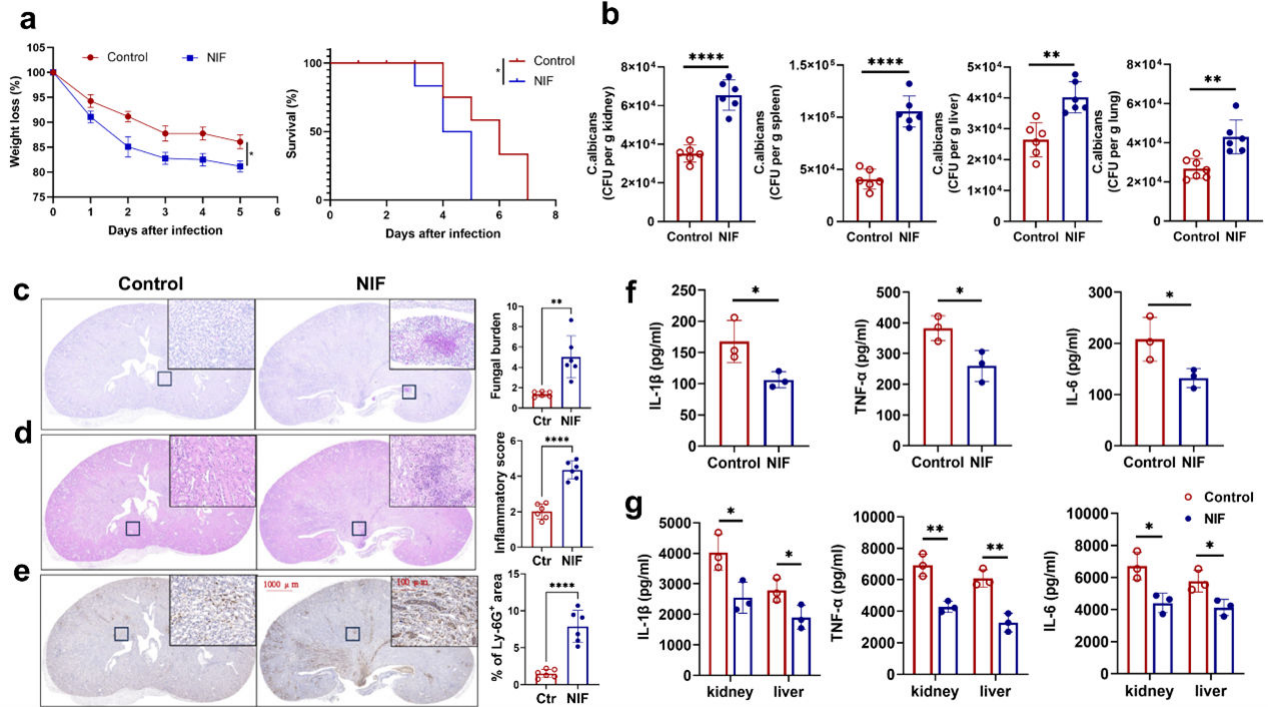
NIF inhibits killing of *C. albicans* by immune system *in vivo*. (**a**) Weight loss and survival of control and NIF (*n* = 12) group mice (6–8-week-old) after infection with *C. albicans* strain SC5314 (50 µL, 4 × 10^6^ CFU/mL) (left: *P* = 0.028, right: *P* = 0.022). (**b**) Statistics of *C. albicans* in the kidneys, livers, lungs, and spleens of control and NIF group mice infected with *C. albicans* for 5 days, evaluated by serial dilution of homogenized tissues and presented as CFU per gram of the indicated tissue (*n* = 6 per group) (from left to right: 1, *P* < 0.0001; 2, *P* < 0.0001; 3, *P* = 0.0061; 4, *P* = 0.0052). (**c**) Representative images of hematoxylin-eosin (HE)-stained kidney sections of control and NIF group mice 5 days after systemic *C. albicans* infection and obtained inflammatory score based on tissue destruction and renal immune cell infiltration (*n* = 6 per group) (*P* = 0.0016). (**d**) Representative images of periodic acid-Schiff (PAS)-stained kidney sections of control and NIF group mice 5 days after *C. albicans* infection and obtained score based on fungal load (*n* = 6 per group) (*P* < 0.0001). (**e**) Representative images of kidney sections stained for the neutrophil marker Ly-6G^+^ and quantification of kidney area scored as Ly-6G^+^ 5 days after infection of control and NIF group mice with *C. albicans* (*n* = 6 per group) (*P* < 0.0001). Scale bars: 1000 µm, 100 µm (c–e). (**f**) ELISA analysis of the IL-1β, IL-6, and TNF-α from the serum of the control and NIF group mice 24 h after infection with *C. albicans* (*n* = 3 per group) (left: *P* = 0.017; middle: *P* = 0.026; right: *P* = 0.022). (**g**) ELISA analysis of IL-1β, IL-6, and TNF-α from the supernatant of the organ of the control and NIF group mice 5 days after systemic *C. albicans* infection (*n* = 3 per group) (left: *P* = 0.015, *P* = 0.036; middle: *P* = 0.0034, *P* = 0.0058; right: *P* = 0.029, *P* = 0.041). Data are shown as mean ± SD in b–g. *P < 0.05; **P < 0.01; ****P < 0.0001 (two-way analysis of variance and log-rank [Mantel-Cox] test [a] or Student’s *t*-test [b–g]).

To confirm the *in vivo* function of NIF in inhibiting the antifungal immune response, mice were pretreated via intraperitoneal injection 30 min prior to infection, with the control group receiving saline solution and the experimental group administered NIF. Subsequently, all animals were intravenously challenged with a moderate inoculum of *C. albicans*. The results showed that the mice in the NIF group lost a significant amount of body weight and exhibited a lower survival after infection with *C. albicans* (Fig. 4a). To further elucidate the role of NIF in antifungal immunity, we assessed the fungal load in mice at 5 days post-infection. We found that mice in the NIF group exhibited more *C. albicans* colony-forming units (CFU) in the kidney, spleen, and liver (Fig. 4b). Histopathological analysis of the kidneys showed increased inflammation in the kidneys of mice in the NIF group with large numbers of *C. albicans* yeast cells and hyphae (Fig. 4c and d). In addition, Ly6G immunohistochemistry revealed significantly more neutrophil infiltration in the kidneys of mice in the NIF group than in the control group. This might be due to low neutrophil fungicidal activity, excessive fungal aggregation, and subsequent excessive inflammation, causing massive neutrophil accumulation (Fig. 4e).

In addition, we assessed the innate immune response in mice following *C. albicans* infection. At the early time point of *C. albicans* infection, mice in the NIF group had significantly reduced serum secretion of IL-1β, IL-6, and TNF-α at 24 h post-infection compared with control mice (Fig. 4f). Consistently, the NIF group also had lower levels of IL-1β, IL-6, and TNF-α in the homogenates of kidney and liver after 5 days of infection with *C. albicans* (Fig. 4g).

### Prolonged total hospital stays for patients with fungal infection using dihydropyridine calcium channel blockers

**Fig 5:**
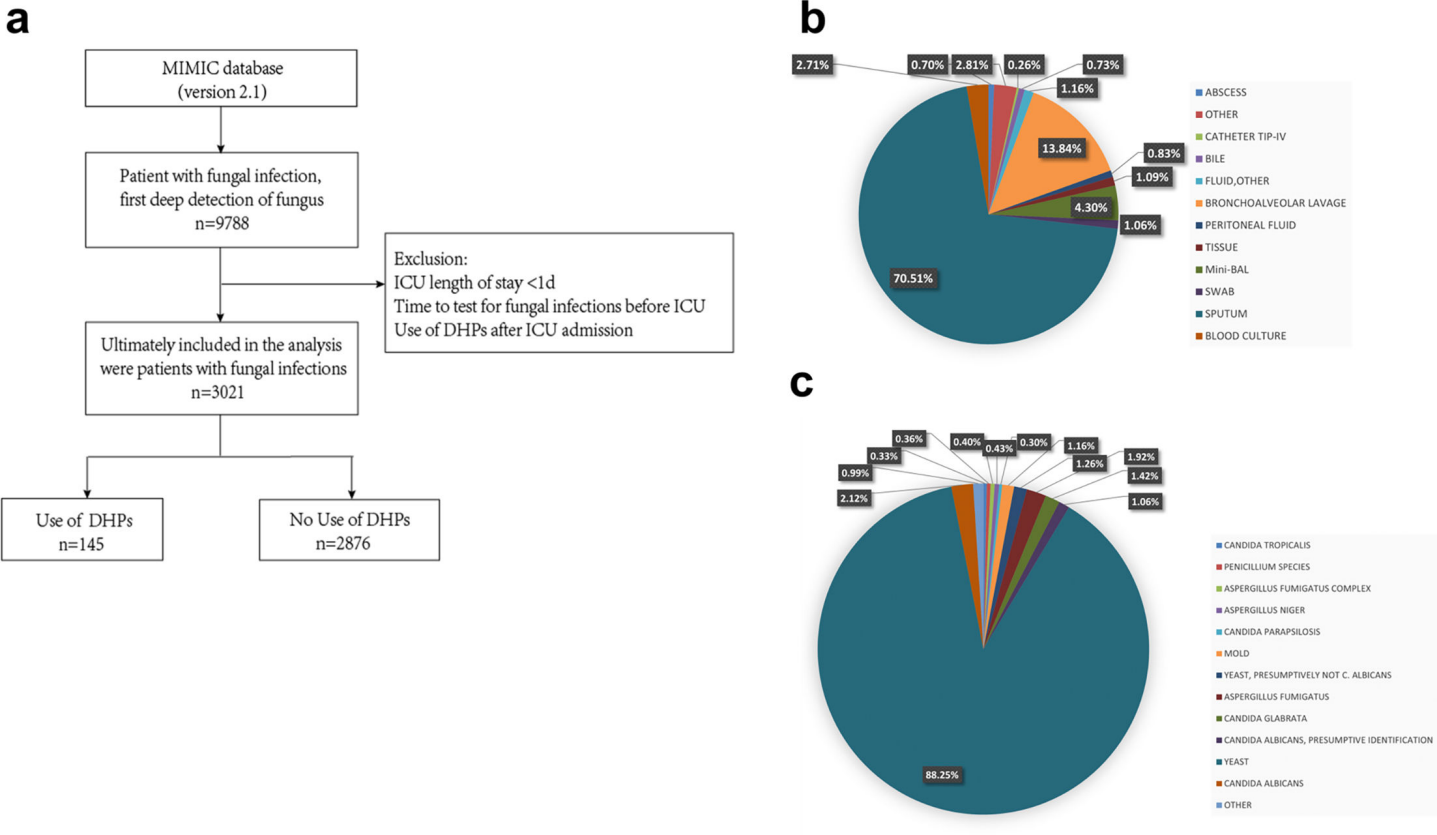
Extraction of patients with fungal infections from the MIMIC database. (**a**) Cohort selection schema. (**b**) Type distribution of samples tested from patients with fungal infections. (**c**) Type distribution of pathogenic fungi in patients with fungal infections.

We utilized the Critical Care Medical Information Marketplace (MIMIC) database, which is developed and maintained by MIT’s Computational Physiology Laboratory. ICU patients with fungal infection were extracted from the MIMIC-IV 2.0 database using ICD-10 codes (B37), and only patients with the first deep tissue detection of fungi were included, excluding patients with positive respiratory and gastrointestinal fungi tests; a total of 9,788 patients were screened. Based on the exclusion criteria shown in Fig. 5a, 3,021 patients were finally included, that is, 145 individuals who had used dihydropyridine calcium channel blockers (DHPs) and 2,876 individuals who had not used DHPs. Figure 5b illustrates the type of sample for fungal testing. Figure 5c demonstrates the types of fungal infection in patients, with the highest percentage being yeasts.

Basic information about the patient was extracted, including (i) demographic characteristics: age and weight; (ii) vital signs: heart rate, body temperature, blood oxygen saturation (SpO_2_), respiratory rate (bpm), and body temperature; (iii) laboratory results: total bilirubin, anion gap, creatinine, blood urea nitrogen (BUN), white blood cells, glucose, hemoglobin, bicarbonate, creatinine, sodium, partial thromboplastin time (PTT), prothrombin time (PT), and international normalized ratio (INR); (iv) score for severity of illness: GCS, SAPS II, SOFA, and CCI; (v) comorbidities: chronic lung disease, congestive heart failure, diabetes mellitus, kidney disease, malignant cancer, severe liver disease, rheumatic disease, peripheral vascular disease, and cerebrovascular disease. Baseline characteristics of the two groups were compared (Table S1), with no difference in baseline characteristics between the two groups by propensity matching scores (Table 1), and then analyzed in terms of length of stay and mortality between pre-matched and post-matched patients, with the results of the pre-matching analyses showing a prolongation of the total length of stay and no difference in mortality rates in patients using DHPs (Table S2). Post-matching analyses showed prolonged total length of stay and ICU length of stay in patients using DHPs (Table 2).

**TABLE 1:** Baseline characteristics of patients with fungal infections in a propensity-matched cohort

	DHP group *n* = 141	No DHP group *n* = 141	*P*-value
Age/year	68 (59–77)	69 (56–75)	0.559
Weight (kg)	81.0 (69.0–94.2)	79.0 (62.0–91.9)	0.236
Vital signs			
Heart rate	106 (90–119)	108 (91–120)	0.839
Respiratory rate (bpm)	28 (24–35)	28 (25–32)	0.992
Temperature (°C)	37.5 (37.0–38.1)	37.4 (37.0–38.2)	0.741
SpO_2_	92 (88–95)	93 (89–95)	0.307
Laboratory results			
BUN (mg/dL)	29.0 (19.0–46.0)	28.0 (17.0–42.5)	0.319
WBC (×10^9^/L)	15.1 (10.8–21.1)	15.6 (11.4–22.8)	0.499
Hemoglobin (×10^12^/L)	10.4 (9.1–11.9)	10.5 (9.2–12.4)	0.157
Platelet (×10^9^/L)	179 (120–271)	174 (130–257)	0.802
Creatinine (mg/dL)	1.5 (0.9–2.6)	1.3 (0.8–2.3)	0.191
Glucose	177.0 (135.0–222.0)	169.0 (127.5–227.5)	0.233
Calcium	8.5 (8.1–9.1)	8.4 (8.2–9.1)	0.995
PTT (s)	35.5 (28.6–55.4)	36.9 (29.8–63.5)	0.223
PT (s)	15.5 (13.5–18.6)	15.2 (13.3–17.9)	0.735
INR	1.4 (1.2–1.7)	1.4 (1.2–1.6)	0.535
Severity of illness			
GCS	15 (13–15)	15 (14–15)	0.315
SAPS II	44 (36–53)	43 (32–52)	0.214
SOFA	7 (4–10)	6 (3–9)	0.276
CCI	7 (5–9)	7 (5–9)	0.588
Comorbidities *n* (%)			
Chronic lung disease	45 (31.9)	45 (31.9)	1.000
Congestive heart failure	58 (41.1)	58 (41.1)	1.000
Diabetes mellitus	53 (37.6)	56 (39.7)	0.643
Kidney disease	56 (39.7)	49 (34.8)	0.389
Malignant cancer	21 (14.9)	19 (13.5)	0.733
Severe liver disease	8 (5.7)	10 (7.1)	0.627
Rheumatic disease	7 (5.0)	8 (5.7)	0.389
Peripheral vascular disease	31 (22.0)	24 (17.0)	0.294
Cerebrovascular disease	38 (27.0)	37 (26.2)	0.893

**TABLE 2:** Outcomes of patients with fungal infections in a propensity-matched cohort

Primary outcomes	DHP group *n* = 141	No DHP group *n* = 141	*P*-value
7-Day mortality	10 (7.1)	15 (10.6)	0.296
30-Day mortality	37 (26.2)	44 (31.2)	0.358
90-Day mortality	54 (38.3)	61 (43.3)	0.397
ICU mortality	35 (24.8)	33 (23.4)	0.781
In-hospital mortality	38 (27.0)	43 (30.5)	0.511
Length of stay	26.6 (17.7–42.9)	17.3 (9.9–31.8)	<0.001^*a*^
Length of ICU stay	11.1 (6.1–22.6)	9.7 (5.0–16.0)	0.196

^*a*^ Statistically significant difference.

## DISCUSSION

There is currently no study on whether antihypertensive drugs affect fungal infections. In this study, we first screened common antihypertensive drugs and found that NIF inhibited neutrophil killing of *C. albicans* in a dose-dependent pattern. Then, we explored the main mechanism of action to inhibit its killing and found that NIF inhibited the production of ROS and pro-inflammatory factors in PMNs stimulated by the fungi. It was further elucidated that NIF inhibits the production of downstream pro-inflammatory mediators by inhibiting the phosphorylation of NF-κB. Extracellular stimulation triggers IκB phosphorylation and subsequent degradation, leading to NF-κB activation via phosphorylation. The activated NF-κB complex translocates to the nucleus and binds to specific DNA response elements, and its p65 subunit directly regulates the expression of inflammatory response genes. This induces the production of pro-inflammatory cytokines, chemokines, ROS, iNOS, and other immune mediators (21, 27).

Of note, amlodipine, a drug like NIF, does not inhibit neutrophil-mediated *C. albicans* killing. Though it blocks Ca²^+^ entry into neutrophils, a study shows it enhances neutrophil phagocytosis via an unclear mechanism (28). This may explain the different functions of the two drugs.

Finally, we constructed a fungal infection model using C57BL/6 mice. Compared with the control group, NIF-administered mice were more susceptible to systemic *C. albicans* infection, with faster body weight loss, heavier organ fungal loads, more severe renal inflammation, more neutrophil recruitment, and significantly reduced serum and tissue supernatant inflammatory cytokine levels, which suggests that NIF *in vivo* also suppresses the immune system’s response to *C. albicans* killing* in vivo*.

As a Ca²^+^ antagonist, NIF may influence neutrophil migration or respiratory burst function; however, its observed effects appear independent of the calcium channel blockade (8, 29). The precise molecular mechanism remains elusive. In our infection model, NIF-treated mice exhibited heightened renal neutrophil infiltration. We hypothesize that impaired neutrophil fungicidal capacity permits enhanced renal accumulation of *C. albicans*, triggering severe local inflammation that subsequently recruits additional neutrophils. It remains undetermined whether NIF suppresses neutrophil migration, and if so, whether such suppression is insufficient to override the potent chemotactic attraction generated by elevated inflammatory cytokines. These mechanistic nuances warrant further investigation.

Also using the MIMIC-IV database to extract patients with fungal infections for analysis, the length of hospitalization was significantly longer in the medicated group, suggesting that NIF may also inhibit the killing of fungi by the immune system *in vivo*. NIF may inhibit NF-κB phosphorylation activation by inhibiting IκB phosphorylation degradation, which in turn inhibits the production of pro-inflammatory cytokines, ROS, and NO, ultimately leading to diminished neutrophil bactericidal capacity. However, these fungal infection cases only cover a subset of *C. albicans* infections, and it is uncertain if the mechanisms studied here apply to other fungal types. Nevertheless, it is clear that NIF does prolong the hospital stay of patients with fungal infections to some extent.

Our study has limitations. First, we did not validate whether amlodipine or verapamil affects ROS/cytokine production or NF-κB phosphorylation. Therefore, our data cannot definitively support that NIF’s mechanism of action is universally unique among all calcium channel blockers. Second, it is unclear whether NIF impacts neutrophil migration. Third, the MIMIC database only includes some *C. albicans* patients, so NIF’s effects on other fungi remain unknown. In addition, NIF inhibited IκB phosphorylation, but its upstream signaling molecules were not examined, so whether NIF directly inhibits IκB phosphorylation or indirectly inhibits NF-κB phosphorylation through other upstream signaling molecules has not been demonstrated.

In summary, this study found that NIF inhibits PMN killing of *C. albicans in vitro* and *in vivo*. We demonstrated that NIF inhibits the killing of *C. albicans* by PMNs and may provide medication advice for patients with clinical hypertension combined with fungal infections.

## Data Availability

The data sets used and/or analyzed during the current study are available from the corresponding author on reasonable request.

## SUPPLEMENTAL MATERIAL

Supplemental Material spectrum.01479-25-s0001.docx Figures S1 and S2; Tables S1 to S4.

ASM does not own the copyrights to Supplemental Material that may be linked to, or accessed through, an article. The authors have granted ASM a non-exclusive, world-wide license to publish the Supplemental Material files. Please contact the corresponding author directly for reuse.
