# Supplementary material for: NIF inhibits the ability of PMNs to produce inflammatory cytokines and reactive oxygen species in response to C. albicans
Source: Microbiol Spectr. 2025 Aug 21;13(10):e01479-25. doi: 10.1128/spectrum.01479-25 (PMC12502607; doi:10.1128/spectrum.01479-25)
Supplement: Supplemental Material — Figures S1 and S2; Tables S1 to S4. [file spectrum.01479-25-s0001.docx]

Supplementary figures


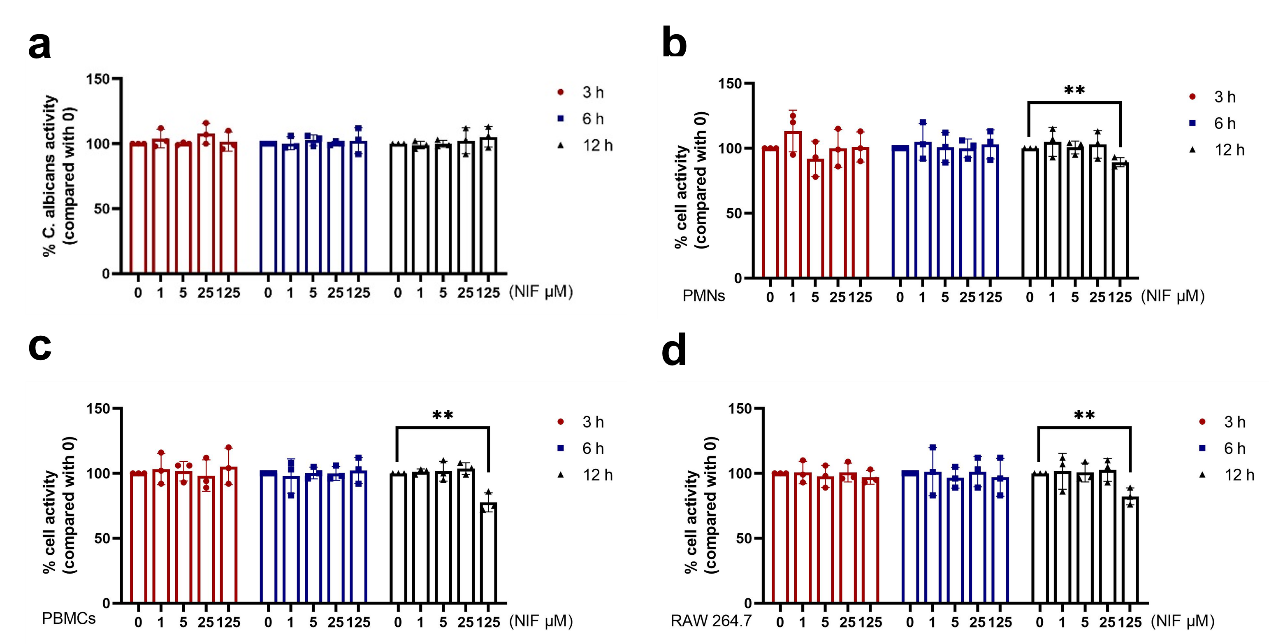


**Supplementary figure 1. Nifedipine has no direct effect on PMNs and Candida albicans.** (a) After co-culture of different concentrations of nifedipine (1, 5, 25, 125 μM) with Candida albicans for 3, 6 and 12 h, the bacterial solution was diluted and coated on plates and the number of colonies was counted. (b, c, d) Different concentrations of nifedipine (1, 5, 25, 125 μM) were co-cultured with PMNs (b), PBMCs (c), and RAW264.7 (d) for 3, 6, and 12 h with CCK8, and the cell activity was detected 2 h later. Data are shown as mean ± SD. ***P*<0.01 (Student’s t-test). Assays were performed in triplicate.


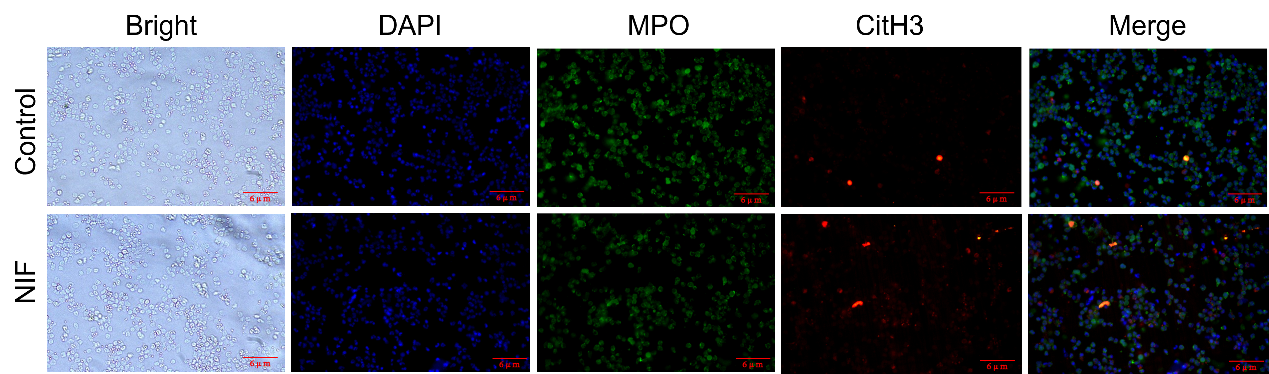


**Supplementary figure 2. Nifedipine does not promote or inhibit the generation of NETs from PMNs.**

Supplementary tables

Supplementary table 1. Baseline characteristics of patients with fungal infections in the unmatched cohort.

|  | **DHPs group**  **n=2876** | **No DHPs group**  **n=145** | ***p*-value** |
| --- | --- | --- | --- |
| Age/year, | 68（59-77） | 66（54-76） | 0.016 |
| Weight (kg) | 80.9（68.9-94.2） | 80.0（66.0-96.0） | 0.710 |
| **Vital signs** |  |  |  |
| Heart rate | 106（90-119） | 112（97-127） | 0.006 |
| Respiratory rate (bpm) | 28（24-35） | 29（25-34） | 0.139 |
| Temperature (℃) | 37.5（37.0-38.2） | 37.5（37.1-38.2） | 0.560 |
| SpO_2_ | 92（88-95） | 92（88-95） | 0.585 |
| **Laboratory results** |  |  |  |
| BUN (mg/dL) | 29.0（19.0-46.0） | 28.0（18.0-46.0） | 0.493 |
| WBC (× 10^9^/L) | 15.1（10.7-21.1） | 15.3（10.5-21.3） | 0.728 |
| Hemoglobin (× 10^12^/L) | 10.4（9.2-11.9） | 10.9（9.4-12.6） | <0.001 |
| Platelet (× 10^9^/L) | 183（121-277） | 165（102-241） | 0.053 |
| Creatinine (mg/dL) | 1.5（0.9-2.6） | 1.3（0.9-2.2） | 0.029 |
| Glucose | 179.0（135.0-222.0） | 159.0（125.0-216.0） | 0.009 |
| Calcium | 8.5（8.1-9.1） | 8.4（8.0-9.0） | 0.069 |
| PTT (s) | 35.5（28.8-55.3） | 36.2（29.8-52.0） | 0.686 |
| PT (s) | 15.5（13.5-18.6） | 15.7（13.6-21.1） | 0.060 |
| INR | 1.4（1.2-1.7） | 1.4（1.2-2.0） | 0.073 |
| **Severity of illness** |  |  |  |
| GCS | 15（12-15） | 15（13-15） | 0.301 |
| SAPS Ⅱ | 44（36-54） | 44（34-55） | 0.531 |
| SOFA | 7（4-10） | 7（5-11） | 0.140 |
| CCI | 7（5-9） | 6（4-8） | <0.001 |
| **Comorbidities n (%)** |  |  |  |
| Chronic lung disease | 45（31.0） | 999（34.7） | 0.361 |
| Congestive heart failure | 58（40.0） | 1007（35.0） | 0.220 |
| Diabetes mellitus | 56（38.6） | 729（25.3） | <0.001 |
| Kidney disease | 59（40.7） | 606（21.1） | <0.001 |
| Malignant cancer | 21（14.5） | 427（14.8） | 0.904 |
| Severe liver disease | 8（5.5） | 348（12.1） | 0.016 |
| Rheumatic disease | 7（4.8） | 119（4.1） | 0.685 |
| Peripheral vascular disease | 34（23.4） | 338（11.8） | <0.001 |
| Cerebrovascular disease | 41（28.3） | 367（12.8） | <0.001 |

SpO_2_, pulse oximeter oxygen saturation; BUN, blood urea nitrogen; WBC, white blood cell; PTT, partial thromboplastin time; PT, prothrombin time; INR, international normalized ratio; GCS, glasgow coma score; SAPS Ⅱ, simplified acute physiology score II; SOFA, sequential organ failure assessment; CCI, charlson comorbidity index.

Supplementary table 2. Outcomes of patients with fungal infections in the unmatched cohort.

| **Primary outcomes** | **DHPs group**  **n=145** | **No DHPs group**  **n=2876** | ***p*-value** |
| --- | --- | --- | --- |
| 7-day mortality | 11（7.6） | 372（12.9） | 0.059 |
| 30-day mortality | 45（31.0） | 1018（35.4） | 0.134 |
| 90-day mortality | 56（38.6） | 1287（44.7） | 0.147 |
| ICU mortality | 36（24.8） | 751（26.1） | 0.731 |
| In-hospital mortality | 39（26.9） | 959（33.3） | 0.107 |
| Length of stay | 26.1（17.5-42.9） | 16.8（9.7-26.8） | <0.001* |
| Length of ICU stay | 11.0（5.5-21.9） | 9.1（4.7-15.8） | 0.008* |

Supplementary table 3. Reagents

| **reagents** | **brand name** | **product number** |
| --- | --- | --- |
| Dextran sulfate | MillporeSigma | 9011-18-1 |
| Ficoll | Cytiva | 17144003 |
| Lysing buffer | BD BioSciences | 555899 |
| PBS | Solarbio | P1022 |
| DMEM | BasalMedia | H211104 |
| FBS | Gibico | A5669701 |
| 1640 | BasalMedia | L211103 |
| Modified Giemsa Staining Solution | Beyotime | C0131 |
| Amphotericin B | Topscience | T1067 |
| Yeast Peptone Dextrose Agar | Solarbio | LA0220 |
| Crystal Violet Staining Solution | Beyotime | C0121 |
| Yeast Peptone Dextrose Broth | Solarbio | LA5010 |
| Trypsin-EDTA Solution | Beyotime | C0201-100 |
| Cellsaving | NCM biotech | C40100 |
| Trypan Blue | Beyotime | C0011-1 |
| RIPA | NCM biotech | WB3100 |
| SDS-PAGE Sample Loading Buffer | Beyotime | P0015L |
| SDS | CST | 20533 |
| Tris-Glycine SDS Electrophoresis Buffer | NCM biotech | WB52001 |
| Transfer Buffer | NCM biotech | WB52002 |
| Triton X-100 | Beyotime | P0096 |
| TBS | Beyotime | ST661 |
| Tween-20 | Solarbio | T8220 |
| NcmECL Ultra | NCM biotech | P10100 |
| Methanol | Sense Chemical | 67-56-1 |
| Ethyl Alcohol | Sense Chemical | 64-17-5 |
| Formaldehyde | Macklin | M813895 |
| 4% PFA Fix Solution | Beyotime | P0099 |
| Penicillin-Streptomycin Solution | Beyotime | C0222 |
| CCK8 | MCE | HY-K0301 |
| DMSO | MCE | HY-Y0320 |
| Nifedipine | Topscience | T1146 |
| DAPI | Abcam | ab104139 |
| NF-κB-IN-1 | MCE | HY-138537 |
| skimmed milk | Biosharp | BS102 |
| PrimeScript™ RT Master Mix | TaKaRa | RR036Q |

Supplementary table 4. Antibodies.

| **antibodies** | **brand name** | **product number** |
| --- | --- | --- |
| Phospho-JNK Recombinant antibody | Proteintech | 80024-1-RR |
| ERK1/2 Rabbit mAb | ABclonal | A4782 |
| Beta Actin Recombinant antibody | Proteintech | 81115-1-RR |
| Phospho-IκBαRabbit mAb | CST | #2859 |
| HRP-conjugated GAPDH Monoclonal antibody | Proteintech | HRP-60004 |
| IκBαRabbit mAb | ABclonal | A19714 |
| P44/42 MAPK (ERK1/2) Rabbit mAb | CST | #4695 |
| Goat Anti-Rabbit IgG | Abcam | Ab6721 |
| Phospho-Syk Rabbit mAb | CST | #2710 |
| Syk Rabbit mAb | ABclonal | A2123 |
| ERK1/2 Polyclonal antibody | Proteintech | 11257-1-AP |
| Phospho-ERK1/2 Rabbit PolyAb | Proteintech | 28733-1-AP |
| JNK Rabbit mAb | ABclonal | A0288 |
| Phospho-SAPK/JNK Rabbit mAb | CST | #4668 |
| Phospho-p44/42 MAPK (ERK1/2) Rabbit mAb | CST | #4370 |
| Phospho-IkB Recombinant antibody | Proteintech | 42349-1-RR |

**Method**

**Cell viability assay**

Viability assessment was performed via CCK-8 assay (Dojindo Laboratories, Japan). Cells were seeded in 96-well plates at 5×10³ cells/well (100 μL complete DMEM per well). Post-treatment, 10 μL CCK-8 solution was introduced into each well, followed by 2-hour incubation at 37 °C under 5% CO₂ humidified conditions. Optical density measurements were recorded at 450 nm using a microplate reader.
